# Association between (ΔPaO_2_/FiO_2_)/PEEP and in-hospital mortality in patients with COVID-19 pneumonia: A secondary analysis

**DOI:** 10.1371/journal.pone.0304518

**Published:** 2024-05-31

**Authors:** Youli Chen, Huangen Li, Jinhuang Lin, Zhiwei Su, Tianlai Lin

**Affiliations:** Intensive Care Unit, Fujian Medical University Affiliated First Quanzhou Hospital, Quanzhou, Fujian, PR China; NMC Royal Hospital / The National Research Centre, UNITED ARAB EMIRATES

## Abstract

**Background:**

The arterial pressure of oxygen (PaO_2_)/inspiratory fraction of oxygen (FiO_2_) is associated with in-hospital mortality in patients with Coronavirus Disease 2019 (COVID-19) pneumonia. ΔPaO_2_/FiO_2_ [the difference between PaO_2_/FiO_2_ after 24 h of invasive mechanical ventilation (IMV) and PaO_2_/FiO_2_ before IMV] is associated with in-hospital mortality. However, the value of PaO_2_ can be influenced by the end-expiratory pressure (PEEP). To the best of our knowledge, the relationship between the ratio of (ΔPaO_2_/FiO_2_)/PEEP and in-hospital mortality remains unclear. This study aimed to evaluate their association.

**Methods:**

The study was conducted in southern Peru from April 2020 to April 2021. A total of 200 patients with COVID-19 pneumonia requiring IMV were included in the present study. We analyzed the association between (ΔPaO_2_/FiO_2_)/PEEP and in-hospital mortality by Cox proportional hazards regression models.

**Results:**

The median (ΔPaO_2_/FiO_2_)/PEEP was 11.78 mmHg/cmH_2_O [interquartile range (IQR) 8.79–16.08 mmHg/cmH_2_O], with a range of 1 to 44.36 mmHg/cmH_2_O. Patients were divided equally into two groups [low group (< 11.80 mmHg/cmH_2_O), and high group (≥ 11.80 mmHg/cmH_2_O)] according to the (ΔPaO_2_/FiO_2_)/PEEP ratio. In-hospital mortality was lower in the high (ΔPaO_2_/FiO_2_)/PEEP group than in the low (ΔPaO_2_/FiO_2_)/PEEP group [18 (13%) vs. 38 (38%)]; hazard ratio (HR), 0.33 [95% confidence intervals (CI), 0.17–0.61, P < 0.001], adjusted HR, 0.32 (95% CI, 0.11–0.94, P = 0.038). The finding that the high (ΔPaO_2_/FiO_2_)/PEEP group exhibited a lower risk of in-hospital mortality compared to the low (ΔPaO_2_/FiO_2_)/PEEP group was consistent with the results from the sensitivity analysis. After adjusting for confounding variables, we found that each unit increase in (ΔPaO_2_/FiO_2_)/PEEP was associated with a 12% reduction in the risk of in-hospital mortality (HR, 0.88, 95%CI, 0.80–0.97, P = 0.013).

**Conclusions:**

The (ΔPaO_2_/FiO_2_)/PEEP ratio was associated with in-hospital mortality in patients with COVID-19 pneumonia. (ΔPaO_2_/FiO_2_)/PEEP might be a marker of disease severity in COVID-19 patients.

## Introduction

Respiratory failure is a common complication in patients with severe coronavirus disease 2019 (COVID-19). It is a common reason for the need for invasive mechanical ventilation (IMV) and a significant contributor to mortality among individuals with COVID-19 [1]. Studies reported that a proportion of COVID-19 patients (23.6% to 33.1%) required IMV [2,3]. In addition, the mortality rate of COVID-19 patients requiring IMV was approximately 28.6% to 60.4% [3,4]. The arterial pressure of oxygen (PaO_2_)/inspiratory fraction of oxygen (FiO_2_) was both an important indicator of tracheal intubation and a correlate of mortality. Previous studies focused on the relationship between PaO_2_/FiO_2_ and prognosis at the time of admission to the hospital or intensive care unit (ICU) [5,6]. Their studies showed that lower PaO_2_/FiO_2_ was associated with increased in-hospital mortality in COVID-19 patients [7,8]. However, a study by Arnold-Day C et al. [9] showed that PaO_2_/FiO_2_ was not an independent risk factor for death in patients requiring IMV. Another study by Miguel et al. [10] showed that there was no association between PaO_2_/FiO_2_ before IMV and mortality. Therefore, PaO_2_/FiO_2_ might not be a good assessment of prognosis in COVID-19 patients requiring IMV. Miguel et al. also found that the difference between PaO_2_/FiO_2_ after 24 h of IMV and PaO_2_/FiO_2_ before IMV (ΔPaO_2_/FiO_2_) was associated with in-hospital mortality. However, the value of PaO_2_ could be influenced by end-expiratory pressure (PEEP), and patients with higher PEEP might have more severe lung injury than those with lower PEEP for the same ΔPaO_2_/FiO_2_ [11]. Palanidurai S et al. [11] created the (PaO_2_/FiO_2_)/PEEP ratio and found that (PaO_2_/FiO_2_)/PEEP ratio was associated with mortality in patients with acute respiratory distress syndrome. In this study, we aimed to evaluate the relationship between (ΔPaO_2_/FiO_2_)/PEEP and in-hospital mortality in patients with COVID-19 pneumonia.

## Materials and methods

### Study design and participants

This study assessed a retrospective single-center cohort study conducted by Miguel et al [10] from April 2020 to April 2021 in southern Peru. The study reanalyzed 200 patients with COVID-19 pneumonia requiring IMV treatment. This was a secondary analysis study. Mechanical ventilation management strategies and prone position ventilation management strategies for this cohort were described in full previously [10]. The study was approved by the Ethics Committee of Faculty of Health Sciences of the Private University of Tacna (identification code: N391-2021-UPT/FACSA-D). As this was an observational study, and the data were anonymized, the requirement for informed consent was waived. Data were freely extracted from Miguel et al. [10] (https://www.ncbi.nlm.nih.gov/pmc/articles/PMC9756861/bin/peerj-10-14290-s004.xls).

### Stratification definitions

High white blood cells: > 10 × 10^9^/L. High lymphocyte: > 1 × 10^9^/L. High platelets: > 100 × 10^9^/L. High procalcitonin: > 0.5 ng/ml. High C-reactive protein: > 100 mg/L. High alanine aminotransferase: > 40 U/L. High aspartate aminotransferase: > 40 U/L. High creatinine phosphokinase: > 200 U/L. High creatinine phosphokinase-MB: > 25 U/L [12].

High sequential organ failure assessment score: > 4 [13].

High plateau pressure after 24 h of IMV: > 30 cmH_2_O. High driving pressure after 24 h of IMV: > 15 cmH_2_O [10].

(ΔPaO_2_/FiO_2_)/PEEP: the difference between PaO_2_/FiO_2_ after 24 h of IMV and PaO_2_/FiO_2_ before IMV)/PEEP.

### Statistical analysis

Continuous data were presented as mean ± standard deviation (SD) if normally distributed, and median [interquartile range (IQR)], if data were non-normal. Categorical variables were presented as frequency and percentages (n; %). The patients were divided equally into two groups [low group (< 11.80 mmHg/cmH_2_O), and high group (≥ 11.80 mmHg/cmH_2_O)] according to (ΔPaO_2_/FiO_2_)/PEEP. The Kruskal-Wallis test was used for the comparison of continuous variables. Comparisons of categorical variables were performed using the chi-square test or Fisher’s exact probability test. To explore the relationship between (ΔPaO_2_/FiO_2_)/PEEP and in-hospital mortality, we performed univariate and multivariate analyses based on the Cox proportional hazards model. In multivariate analysis, we present results for both unadjusted and fully adjusted analytical models. To maximize statistical power and remove bias, we used multiple imputation (MI). To compare in-hospital mortality in the different groups, subgroup analyses were performed using a stratified Cox proportional hazards model. The effects of (ΔPaO_2_/FiO_2_)/PEEP on in-hospital mortality were assessed using Kaplan-Meier curves (log-rank test). We applied a smooth curve technique to estimate the shape between (ΔPaO_2_/FiO_2_)/PEEP and in-hospital mortality by restricted cubic spline regression. Data were analyzed using EmpowerStats (www.empowerstats.com, X&Y solutions, Boston, Massachusetts, USA) and R software version 3.6.1 (http://www.r-project.org). A two-tailed p-value of < 0.05 was considered statistically significant.

## Results

### Patient and baseline characteristics

Demographic and clinical characteristics of patients were shown in Table 1 according to (ΔPaO_2_/FiO_2_)/PEEP. The mean age of patients was 54.29 ± 12.20 years. Among 200 patients, 42 (21%) were female and 158 (79%) were male. There was a statistical difference in comorbidities between the two groups, with chronic kidney disease and heart failure being more common comorbidities in the low (ΔPaO_2_/FiO_2_)/PEEP group (chronic kidney disease, 8% vs. 0, P = 0.007; heart failure, 10% vs. 0, P = 0.001). Lung damage on computed tomography and platform pressure levels were higher in the low (ΔPaO_2_/FiO_2_)/PEEP group (lung damage on computed tomography > 50%, 73% vs. 52%, P = 0.002; platform pressure levels > 30 cmH_2_O, 33% vs. 20%, P = 0.037). Sepsis, septic shock, acute kidney failure, and renal replacement therapy were also more common in the low (ΔPaO_2_/FiO_2_)/PEEP group (sepsis, 80% vs 63%, P = 0.008; septic shock, 37% vs. 21%, P = 0.013; acute kidney failure, 22% vs. 7%, P = 0.003, renal replacement therapy, 12% vs. 3%, P = 0.016). We found that PaO_2_/FiO_2_ after 24 h of IMV were lower and PEEP was higher in the low (ΔPaO_2_/FiO_2_)/PEEP group compared with the high (ΔPaO_2_/FiO_2_)/PEEP group (197.85 ± 55 vs. 301.49 ± 71.75, P < 0.001). There was no difference in PaO_2_/FiO_2_ before entering IMV between the two groups (100.65 ± 43.62 vs. 99.81 ± 34.18, P = 0.880).

**Table 1:** Demographic and clinical characteristics of patients according to (ΔPaO_2_/FiO_2_)/PEEP.

Variable	Low (< 11.80)	High (≥ 11.80)	χ^2^	p-Value
Number	100	100		
Female	20 (20%)	22 (22%)	0.121	0.728
Age (years)			0.030	0.861
< 65	79 (79%)	80 (80%)		
≥ 65	21 (21%)	20 (20%)		
Obesity	61 (61%)	57 (57%)	0.331	0.565
Hypertension	31 (31%)	22 (22%)	2.079	0.149
Diabetes	22 (22%)	21 (21%)	0.030	0.863
Chronic renal insufficiency	8 (8%)	0 (0)	-	0.007
Heart failure	10 (10%)	0 (0)	10.526	0.001
Asthma	12 (12%)	18 (18%)	1.142	0.235
Immunosuppression	11 (11%)	7 (7.07%)	0.934	0.334
White blood cells (×10^9^/L)			0.731	0.393
≤ 10	53 (53%)	59 (59%)		
> 10	47 (47%)	41 (41%)		
Lymphocytes (×10^9^/L)			0.026	0.873
≤ 1	73 (73%)	74 (74%)		
> 1	27 (27%)	26 (26%)		
Platelets (×10^9^/L)			2.922	0.087
≤ 300	62 (62%)	50 (50%)		
> 300	38 (38%)	50 (50%)		
C-reactive protein (mg/L)			1.027	0.311
≤ 100	32 (33.33%)	38 (40.43%)		
> 100	64 (66.67%)	56 (59.57%)		
Procalcitonin (ng/mL)			3.128	0.077
≤ 0.5	55 (75.34%)	55 (87.30%)		
> 0.5	18 (24.66%)	8 (12.70%)		
Alanine aminotransferase (U/L)			0.226	0.635
≤ 40	26 (26%)	29 (29%)		
> 40	74 (74%)	71 (71%)		
Aspartate aminotransferase (U/L)			0.546	0.460
≤ 40	33 (33%)	38 (38%)		
> 40	67 (67%)	62 (62%)		
Creatinine phosphokinase-Total (U/L)			1.602	0.206
≤ 200	59 (62.77%)	63 (71.59%)		
> 200	35 (37.23%)	25 (28.41%)		
Creatinine phosphokinase-MB (U/L)			0.078	0.780
≤ 25	45 (46.88%)	45 (48.91%)		
> 25	51 (53.12%)	47 (51.09%)		
Lung damage on computed tomography			9.408	0.002
≤ 50%	27 (27%)	48 (48%)		
> 50%	73 (73%)	52 (52%)		
Sequential organ failure assessment			1.962	0.161
≤ 4	61 (61.62%)	71 (71%)		
> 4	38 (38.38%)	29 (29%)		
Antibiotics	99 (99%)	98 (98%)	-	1
Corticosteroids	98 (98%)	96 (96.97%)	-	0.683
Colchicine	32 (32%)	20 (20%)	3.742	0.053
Tocilizumab	14 (14%)	12 (12%)	0.177	0.674
Renal replacement therapy	12 (12%)	3 (3%)	5.834	0.016
Sepsis	80 (80%)	63 (63%)	7.091	0.008
Septic shock	37 (37%)	21 (21%)	6.217	0.013
Acute kidney failure	22 (22%)	7 (7%)	9.074	0.003
Arrhythmia	9 (9.09%)	4 (4%)	2.112	0.146
Pneumonia associated with IMV	26 (26%)	18 (18%)	1.865	0.172
Catheter-associated bacteremia	6 (6%)	5 (5%)	0.096	0.756
Plateau pressure 24 h after IMV (cmH_2_O)			4.338	0.037
≤ 30	67 (67%)	80 (80%)		
> 30	33 (33%)	20 (20%)		
Driving pressure 24 h after IMV (cmH_2_O)			1.025	0.311
≤ 15	36 (36%)	43 (43%)		
> 15	64 (64%)	57 (57%)		
Tidal volume 24 h after IMV (ml)	459.47 ± 82.28	474.27 ± 72.53	t = -1.349	0.179
PaO_2_/FiO_2_ before IMV (mmHg)	100.65 ± 43.62	99.81 ± 34.18	t = 0.151	0.880
PaO_2_/FiO_2_ 24 h after IMV (mmHg)	197.85 ± 55	301.49 ± 71.75	t = -11.464	<0.001
PEEP 24 h after IMV (cmH_2_O)	12.70 ± 2	11.69 ± 1.65	t = 3.893	<0.001
IMV LOS (days)	11 (7–19)	8.50 (5–13.25)	z = -2.297	0.023
ICU LOS (days)	11 (7–17.25)	9 (5–14)	z = -1.886	0.061
Hospital LOS (days)	20 (15–28)	20 (13–29)	z = -0.223	0.822
In-hospital mortality	38 (38%)	13 (13%)	16.450	<0.001

IMV, invasive mechanical ventilation; PEEP, end-expiratory pressure; ICU, intensive care unit; LOS, length of stay.

Data on the immunosuppression, sequential organ failure assessment, corticosteroids, and arrhythmia were missing for 1 patient, on the C-reactive protein for 10 patients, on the procalcitonin for 64 patients, on the creatinine phosphokinase-Total for 18 patients, on the creatinine phosphokinase-MB for 12 patients.

The duration of mechanical ventilation was longer in the low (ΔPaO_2_/FiO_2_)/PEEP group compared with the high (ΔPaO_2_/FiO_2_)/PEEP group [11 (7–19) vs. 8.50 (5–13.25)]. Mortality was higher in the low (ΔPaO_2_/FiO_2_)/PEEP group compared to the high (ΔPaO_2_/FiO_2_)/PEEP group [38 (38%) vs. 13 (13%)] (Table 1).

### Univariate analysis of mortality

As reported in Table 2, in the univariate analysis, we found that diabetes, chronic renal insufficiency, heart failure, immunosuppression, lung damage on computed tomography > 50%, sequential organ failure assessment score > 4, tocilizumab, renal replacement therapy, septic shock, acute kidney failure, pneumonia associated with IMV, and plateau pressure 24 h after IMV were associated with higher in-hospital death risk. Based on clinical and statistical reasons (S1 Table), we chose confounding factors including sex, age, obesity, diabetes, chronic renal insufficiency, heart failure, asthma, immunosuppression, platelets, C-reactive protein, procalcitonin, alanine aminotransferase, aspartate aminotransferase, lung damage on computed tomography, sequential organ failure assessment score, tocilizumab, renal replacement therapy, sepsis, septic shock, acute kidney failure, pneumonia associated with IMV, plateau pressure 24 h after IMV, and driving pressure 24 h after IMV.

**Table 2:** Univariate analysis of prognostic factors.

Variable	In-hospital mortality HR (95% CI)	P-value
Sex		
Female	1.0	
Male	0.90 (0.45, 1.81)	0.770
Age (year)		
< 65	1.0	
≥ 65	1.35 (0.76, 2.41)	0.311
Obesity		
No	1.0	
Yes	1.16 (0.66, 2.03)	0.605
Hypertension		
No	1.0	
Yes	1.21 (0.68, 2.15)	0.525
Diabetes		
No	1.0	
Yes	1.91 (1.04, 3.49)	0.036
Chronic renal insufficiency		
No	1.0	
Yes	6.86 (3.06, 15.38)	<0.001
Heart failure		
No	1.0	
Yes	2.85 (1.32, 6.12)	0.007
Asthma		
No	1.0	
Yes	1.27 (0.66, 2.44)	0.469
Immunosuppression		
No	1.0	
Yes	3.54 (1.80, 6.96)	<0.001
White blood cells (×10^9^/L)		
≤ 10	1.0	
> 10	1.46 (0.83, 2.56)	0.184
Lymphocytes (×10^9^/L)		
≤ 1	1.0	
> 1	1.39 (0.75, 2.59)	0.296
Platelets (×10^9^/L)		
≤ 300	1.0	
> 300	0.57 (0.31, 1.03)	0.064
C-reactive protein (mg/L)		
≤ 100	1.0	
> 100	1.87 (0.95, 3.67)	0.068
Procalcitonin (ng/mL)		
≤ 0.5	1.0	
> 0.5	1.26 (0.61, 2.58)	0.535
Alanine aminotransferase (U/L)		
≤ 40	1.0	
> 40	0.63 (0.35, 1.12)	0.114
Aspartate aminotransferase (U/L)		
≤ 40	1.0	
> 40	1.01 (0.56, 1.80)	0.986
Creatinine phosphokinase-Total (U/L)		
≤ 200	1.0	
> 200	1.61 (0.91, 2.84)	0.104
Creatinine phosphokinase-MB (U/L)		
≤ 25	1.0	
> 25	1.11 (0.63, 1.97)	0.712
Lung damage on computed tomography		
≤ 50%	1.0	
> 50%	2.89 (1.23, 6.82)	0.015
Sequential organ failure assessment		
≤ 4	1.0	
> 4	3.20 (1.78, 5.76)	<0.001
Corticosteroids		
No	1.0	
Yes	0.91 (0.12, 6.61)	0.924
Colchicine		
No	1.0	
Yes	1.33 (0.75, 2.36)	0.335
Tocilizumab		
No	1.0	
Yes	2.53 (1.31, 4.91)	0.006
Renal replacement therapy		
No	1.0	
Yes	3.75 (1.98, 7.14)	<0.001
Sepsis		
No	1.0	
Yes	1.55 (0.69, 3.47)	0.286
Septic shock		
No	1.0	
Yes	2.91 (1.60, 5.29)	<0.001
Acute kidney failure		
No	1.0	
Yes	3.25 (1.85, 5.71)	<0.001
Arrhythmia		
No	1.0	
Yes	1.79 (0.85, 3.75)	0.122
Pneumonia associated with IMV		
No	1.0	
Yes	3.89 (2.21, 6.84)	<0.001
Catheter-associated bacteremia		
No	1.0	
Yes	1.40 (0.55, 3.52)	0.481
Plateau pressure 24 h after IMV (cmH_2_O)		
≤ 30	1.0	
> 30	2.23 (1.28, 3.88)	0.004
Driving pressure 24 h after IMV (cmH_2_O)		
≤ 15	1.0	
> 15	1.46 (0.81, 2.64)	0.210

HR, hazard ratio; CI, confidence interval; IMV, invasive mechanical ventilation.

### Sensitivity analyses

We conducted a stratified analysis according to baseline characteristics. In subgroup analyses (Table 3), the high (ΔPaO_2_/FiO_2_)/PEEP group was associated with lower in-hospital mortality in most strata compared with the low (ΔPaO_2_/FiO_2_)/PEEP group. When the sample size was less than 10, we did not perform stratified analyses. As shown in Table 3 and S2 Table, the interaction test was statistically significant in both age and C-reactive protein groups (p < 0.05). In patients aged < 65 years, the risk of death was lower in the high group (HR, 0.18, 95% CI, 0.08–0.45, P = 0.026). The risk of death was higher in the low group among patients with C-reactive protein > 100 mg/L (HR, 3.63, 95%CI, 1.28–10.31, P = 0.007).

**Table 3:** Stratified analysis of (ΔPaO_2_/FiO_2_)/PEEP and in-hospital mortality.

Variable	Total	Death	HR (95% CI)		P for interaction
			Low (< 11.80)	High (≥ 11.80)	
Sex					0.5523
Female	42	10	1.0	0.46 (0.12, 1.79)	
Male	158	41	1.0	0.29 (0.14, 0.60)	
Age (year)					0.0264
< 65	159	33	1.0	0.20 (0.08, 0.48)	
≥ 65	41	18	1.0	0.85 (0.32, 2.26)	
Obesity					0.6244
No	82	21	1.0	0.26 (0.10, 0.68)	
Yes	118	30	1.0	0.37 (0.16, 0.86)	
Hypertension					0.4759
No	147	33	1.0	0.38 (0.18, 0.80)	
Yes	53	18	1.0	0.24 (0.07, 0.85)	
Diabetes					0.4880
No	157	35	1.0	0.35 (0.17, 0.76)	
Yes	43	16	1.0	0.24 (0.08, 0.77)	
Chronic renal insufficiency					-
No	192	44	1.0	0.38 (0.20, 0.72)	
Heart failure					-
No	190	43	1.0	0.36 (0.19, 0.70)	
Asthma					0.7396
No	170	38	1.0	0.33 (0.16, 0.71)	
Yes	30	13	1.0	0.29 (0.09, 0.94)	
Immunosuppression					-
No	181	40	1.0	0.37 (0.19, 0.75)	
Yes	18	11	1.0	0.13 (0.02, 1.05)	
White blood cells (×10^9^/L)					0.0804
≤ 10	112	22	1.0	0.57 (0.24, 1.37)	
> 10	88	29	1.0	0.19 (0.07, 0.50)	
Lymphocytes (×10^9^/L)					0.0550
≤ 1	147	37	1.0	0.45 (0.22, 0.89)	
> 1	53	14	1.0	0.10 (0.01, 0.75)	
Platelets (×109/L)					0.1392
≤ 300	112	36	1.0	0.48 (0.23, 1.01)	
> 300	88	15	1.0	0.16 (0.05, 0.58)	
C-reactive protein (mg/L)					0.0072
≤ 100	70	11	1.0	1.37 (0.39, 4.75)	
> 100	120	37	1.0	0.17 (0.07, 0.44)	
Procalcitonin (ng/mL)					0.5028
≤ 0.5	110	31	1.0	0.27 (0.12, 0.63)	
> 0.5	26	10	1.0	0.22 (0.03, 1.75)	
Alanine aminotransferase (U/L)					0.5016
≤ 40	55	18	1.0	0.45 (0.17, 1.21)	
> 40	145	33	1.0	0.26 (0.11, 0.61)	
Aspartate aminotransferase (U/L)					0.2649
≤ 40	71	17	1.0	0.18 (0.05, 0.63)	
> 40	129	34	1.0	0.43 (0.20, 0.89)	
Creatinine phosphokinase-Total (U/L)					0.7565
≤ 200	122	29	1.0	0.30 (0.13, 0.69)	
> 200	60	29	1.0	0.35 (0.11, 1.11)	
Creatinine phosphokinase-MB (U/L)					0.5275
≤ 25	90	22	1.0	0.22 (0.08, 0.66)	
> 25	98	26	1.0	0.38 (0.16, 0.90)	
Lung damage on computed tomography					0.3173
≤ 50%	75	6	1.0	1.05 (0.19, 5.81)	
> 50%	125	45	1.0	0.32 (0.16, 0.68)	
Sequential organ failure assessment					0.2718
≤ 4	132	17	1.0	0.46 (0.17, 1.26)	
> 4	67	34	1.0	0.26 (0.11, 0.61)	
Corticosteroids					-
Yes	194	50	1.0	0.33 (0.18, 0.63)	
Colchicine					0.5684
No	148	33	1.0	0.29 (0.14, 0.63)	
Yes	52	18	1.0	0.51 (0.17, 1.54)	
Tocilizumab					0.5710
No	174	39	1.0	0.31 (0.15, 0.65)	
Yes	26	12	1.0	0.55 (0.14, 2.08)	
Renal replacement therapy					-
No	185	38	1.0	0.44 (0.22, 0.88)	
Yes	15	13	1.0	0.14 (0.02, 1.11)	
Sepsis					0.8296
No	57	7	1.0	0.26 (0.05, 1.44)	
Yes	143	44	1.0	0.35 (0.17, 0.70)	
Septic shock					0.7716
No	142	17	1.0	0.30 (0.11, 0.87)	
Yes	58	34	1.0	0.41 (0.19, 0.92)	
Acute kidney failure					0.1220
No	171	30	1.0	0.50 (0.24, 1.06)	
Yes	29	21	1.0	0.18 (0.04, 0.79)	
Arrhythmia					-
No	186	42	1.0	0.22 (0.11, 0.47)	
Yes	13	9	1.0	2.56 (0.62, 10.55)	
Pneumonia associated with IMV					0.8398
No	156	21	1.0	0.33 (0.12, 0.89)	
Yes	44	30	1.0	0.32 (0.14, 0.73)	
Catheter-associated bacteremia					-
No	189	46	1.0	0.35 (0.18, 0.67)	
Yes	11	5	1.0	0.14 (0.02, 1.35)	
Plateau pressure 24 h after IMV (cmH_2_O)					0.5694
≤ 30	147	27	1.0	0.30 (0.13, 0.69)	
> 30	53	24	1.0	0.45 (0.17, 1.20)	
Driving pressure 24 h after IMV (cmH_2_O)					0.8875
≤ 15	79	16	1.0	0.28 (0.09, 0.91)	
> 15	121	35	1.0	0.32 (0.15, 0.70)	

HR, hazard ratio, CI; confidence interval; IMV, invasive mechanical ventilation.

### Multivariate analyses of (ΔPaO_2_/FiO_2_)/PEEP and in-hospital mortality

**Fig 1:**
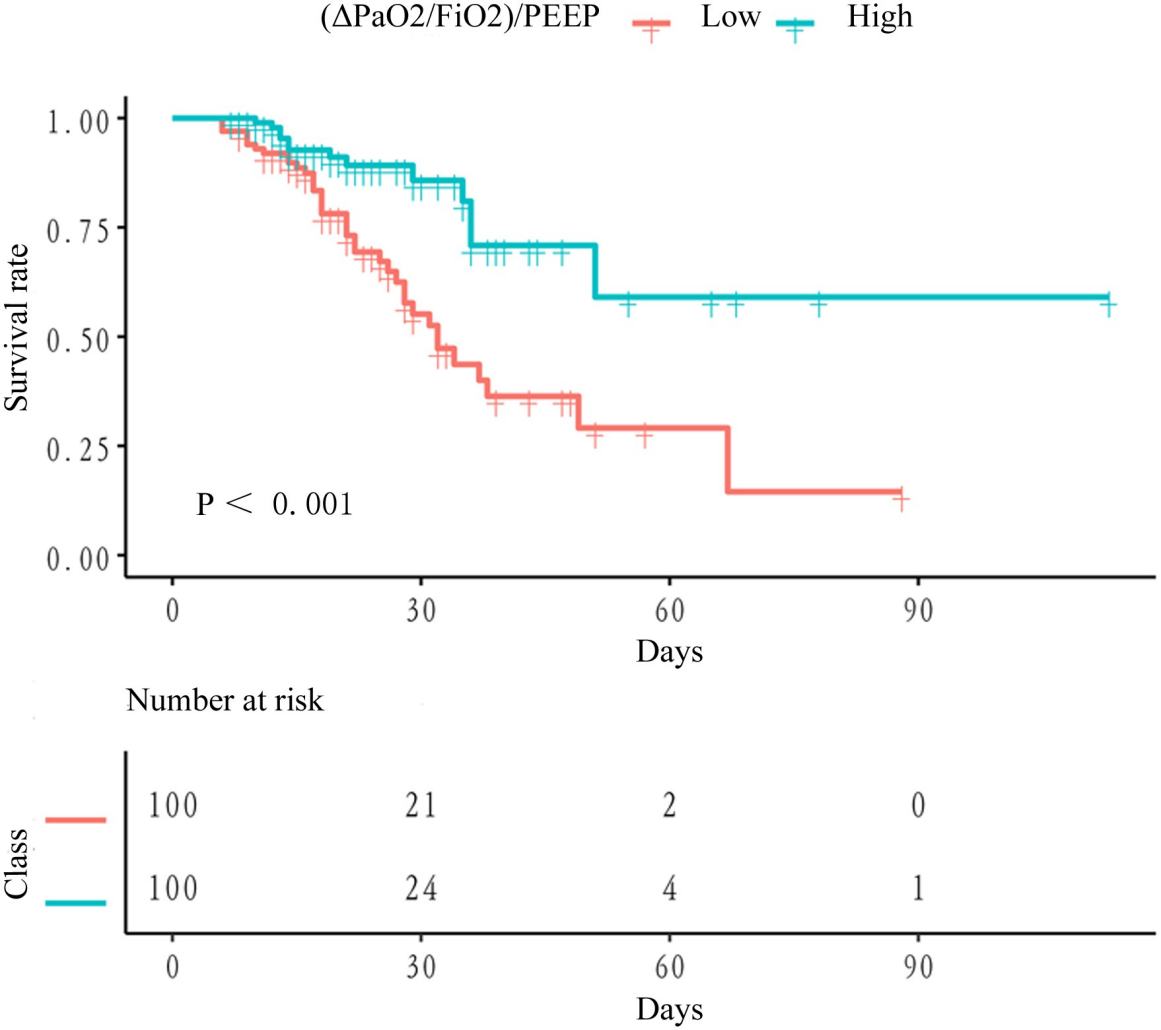
Kaplan-Meier curves for patients in different (ΔPaO_2_/FiO_2_)/PEEP groups.

**Fig 2:**
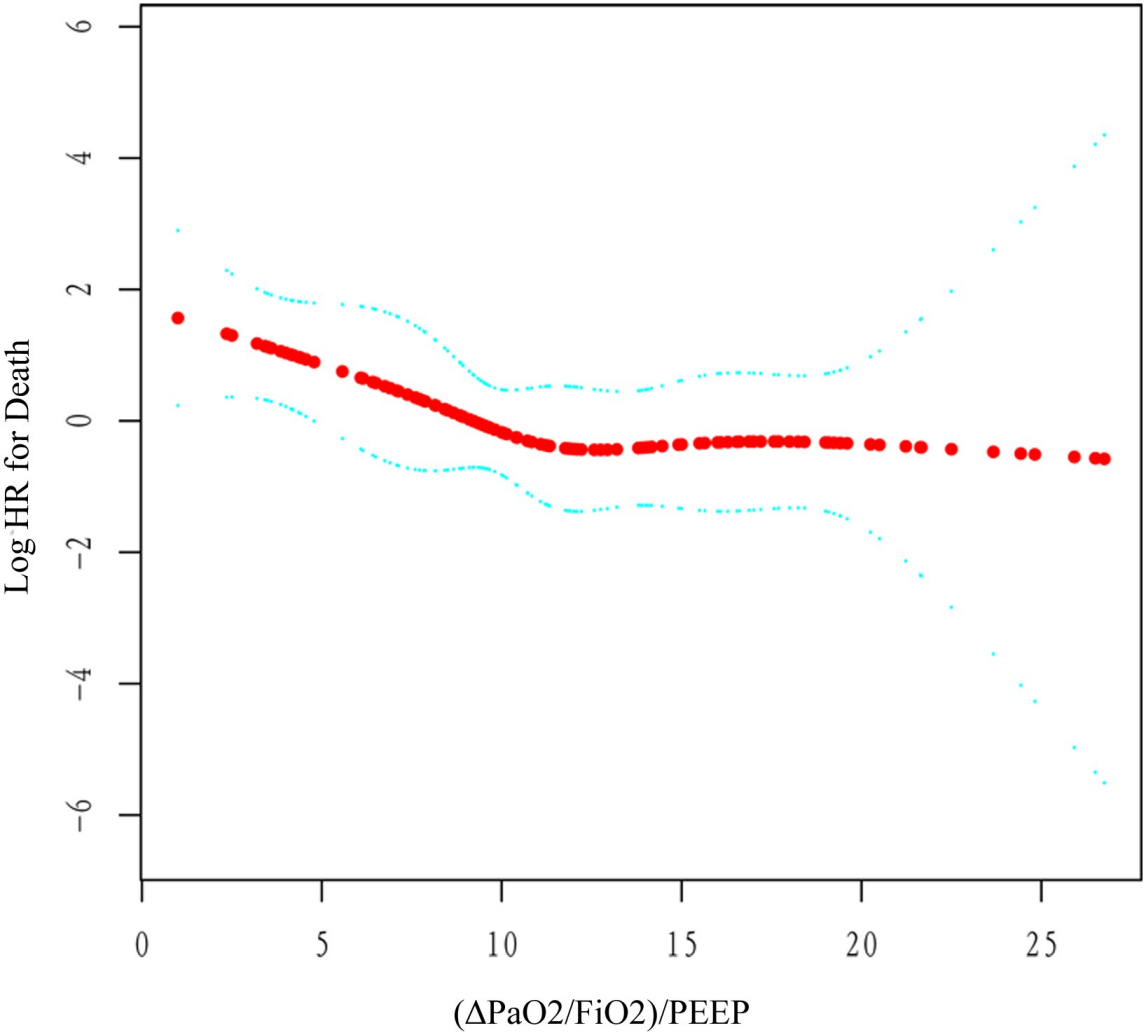
Smoothed curve between (ΔPaO_2_/FiO_2_)/PEEP and in-hospital mortality.

Out of 200 patients, 51 (25.50%) died. The Kaplan-Meier curves for survival rate were shown in Fig 1. The low (ΔPaO_2_/FiO_2_)/PEEP group with the highest mortality (38%) was the reference group (Table 4). As shown in Table 4, the high (ΔPaO_2_/FiO_2_)/PEEP levels group was associated with a 67% risk decrease in death (HR, 0.33, 95%CI, 0.17–0.61, P < 0.001). After adjusting for confounding factors, the relationship was still robust (HR, 0.32, 95%CI, 0.11–0.94, P = 0.038). After adjusting for confounding variables, we found that each unit increase in (ΔPaO_2_/FiO_2_)/PEEP was associated with a 12% reduction in the risk of in-hospital mortality (HR, 0.88, 95%CI, 0.80–0.97, P = 0.013). After excluding outliers (values less than Q1–1.5*IQR or greater than Q3 + 1.5*IQR), where IQR is the interquartile range and Q1 and Q3 are the first and third quartiles, respectively, the risk of death decreased with (ΔPaO2/FiO2)/PEEP [(ΔPaO_2_/FiO_2_)/PEEP < 27.02 cmH_2_O] increasing (Fig 2).

**Table 4:** Cox regression model for In-hospital mortality.

Variable		Unadjusted		Model I		Model II	
		HR(95% CI)	p-value	HR(95% CI)	p-value	HR(95% CI)	p-value
Initial cohort	Low (< 11.80)	1.0		1.0		1.0	
	High (≥ 11.80)	0.33 (0.17, 0.61)	<0.001	0.08 (0.02, 0.31)	<0.001	0.32 (0.11, 0.94)	0.038
	Per (ΔPaO_2_/FiO_2_)/PEEP	0.90 (0.85, 0.95)	<0.001	0.82 (0.74, 0.91)	<0.001	0.88 (0.80, 0.97)	0.013
After MI	Low (< 11.80)	1.0		1.0		1.0	
	High (≥ 11.80)	-	-	0.25 (0.11, 0.59)	0.002	0.36 (0.16, 0.80)	0.013
	Per (ΔPaO_2_/FiO_2_)/PEEP	-	-	0.89 (0.83, 0.96)	0.002	0.90 (0.83, 0.97)	0.009

HR, hazard ratio; CI, confidence interval; MI, multiple imputation.

Model I adjusted for: sex, age (< 65, ≥ 65), obesity, diabetes, chronic renal insufficiency, heart failure, asthma, immunosuppression, platelets (≤ 300, > 300), C-reactive protein (≤ 100, > 100), procalcitonin (≤ 0.5, > 0.5), alanine aminotransferase (≤ 40, > 40), aspartate aminotransferase (≤ 40, > 40), lung damage on computed tomography (≤ 50%, > 50%), sequential organ failure assessment (≤ 4, > 4), tocilizumab, renal replacement therapy, sepsis, septic shock, acute kidney failure, pneumonia associated with IMV, plateau pressure 24 h after IMV (≤ 30, > 30), driving pressure 24 h after IMV (≤ 15, > 15).

Model II adjusted for: sex, age, obesity, diabetes, chronic renal insufficiency, heart failure, asthma, immunosuppression, Platelets, C-reactive protein, procalcitonin, alanine aminotransferase, aspartate aminotransferase, lung damage on computed tomography, sequential organ failure assessment, tocilizumab, renal replacement therapy, sepsis, septic shock, acute kidney failure, pneumonia associated with IMV, plateau pressure 24 h after IMV, driving pressure 24 h after IMV.

Adjusted for sex, age, obesity, diabetes, chronic renal insufficiency, heart failure, asthma, immunosuppression, platelets, C-reactive protein, procalcitonin, alanine aminotransferase, aspartate aminotransferase, lung damage on computed tomography, sequential organ failure assessment, tocilizumab, renal replacement therapy, sepsis, septic shock, acute kidney failure, pneumonia associated with IMV, plateau pressure 24 h after IMV, driving pressure 24 h after IMV.

We also used MI to maximize statistical power and remove bias. The MI was based on five replications and the Markov chain Monte Carlo method in the MI procedure in R to account for missing data on C-reactive protein and Procalcitonin. Results were similar to those of the initial cohort adjusted for potential confounders (Table 4).

## Discussion

In this study, the median duration of mechanical ventilation was 10 days, the median PEEP was 12 cmH_2_O, and the mortality rate was 25%, which was consistent with previous studies [14,15]. Hypertension and obesity were the most common comorbidities in patients with COVID-19 pneumonia, and the majority of patients were male, which was also consistent with previous studies [16].

The PaO_2_/FiO_2_ ratio had some limitations. In patients requiring IMV, PaO_2_/FiO_2_ values could be overestimated on admission to the hospital or ICU [7]. On the other hand, PaO_2_/FiO_2_ values before IMV might be underestimated in emergency situations such as sputum occlusion and respiratory arrest [17]. In addition, the mode of oxygen administration before tracheal intubation, such as nasal cannula oxygenation, mask oxygenation, high-flow oxygen therapy, and noninvasive ventilator-assisted ventilations, might also affect the pre-mechanical ventilation PaO_2_/FiO_2_ values [18].

Patients with significant changes in the difference in PaO_2_/FiO_2_ before and after intubation (ΔPaO_2_/FiO_2_) might not have diffuse alveolar damage [19]. Patients requiring higher PEEP to prevent recurrent alveolar collapse might have combined more severe lung damage [20]. In this study, we found that the low (ΔPaO_2_/FiO_2_)/PEEP group had lower ΔPaO_2_/FiO_2_ values and higher PEEP values compared to the high (ΔPaO_2_/FiO_2_)/PEEP group. After adjusting for potential confounders, the high (ΔPaO_2_/FiO_2_)/PEEP group was associated with a 68% lower risk of death. Previous studies showed that a high percentage of COVID-19 patients admitted to ICU developed cardiac systolic and diastolic dysfunction [21–23]. Although higher PEEP values helped to improve oxygenation, high PEEP values could also lead to impaired right ventricular and hemodynamic function [24]. Patients might require additional fluid and vasopressors. Since higher values of PEEP could affect cardiac function [25], this might partly explain the higher mortality in patients with low (ΔPaO_2_/FiO_2_)/PEEP group. Due to the limitations of the secondary study, data on cardiac insufficiency was not available in the primary dataset. We were unable to further analyze the effect of (ΔPaO_2_/FiO_2_)/PEEP on the prognosis of patients with cardiac insufficiency.

In this study, all patients were placed in the prone position for 48 to 72 continuous hours [10]. To achieve the prone ventilation goal, patients might require high regimens of sedatives, analgesics, and neuromuscular blocking agents. Deep sedation in COVID-19 patients might be associated with several complications such as ventilation-associated pneumonia, prolonged MV duration, and ICU-acquired weakness [26]. These complications might further lead to prolonged ICU and hospitalization and increased mortality [26,27]. However, we were unable to obtain data on analgesic sedatives and neuromuscular blocking agents and therefore could not further analyze their effects on mortality.

### Limitations

This study had some limitations. First, this was a single-center retrospective cohort study to evaluate the association between (ΔPaO_2_/FiO_2_)/PEEP and in-hospital mortality in COVID-19 patients requiring IMV. Thus, the findings might not be generalizable to the general population. Second, this study was a secondary analysis and could not further assess the effect of FiO_2_ on (ΔPaO_2_/FiO_2_). Third, due to limited resources, this study did not adopt an extracorporeal circulation membrane strategy to manage hypoxemia. Fourth, although we adjusted for possible confounding variables, other unmeasured variables might affect the results [28].

## Conclusions

The (ΔPaO_2_/FiO_2_)/PEEP ratio was associated with in-hospital mortality in patients with COVID-19 pneumonia. (ΔPaO_2_/FiO_2_)/PEEP might be a marker of disease severity in COVID-19 patients.

## Supporting information

S1 Table The regression coefficient in the basic model and full model. IMV, invasive mechanical ventilation. These covariates produced over 10% change in the regression coefficient of (ΔPaO_2_/FiO_2_)/PEEP and were adjusted in multivariate analysis when added to the basic model or removed from the full model.
(DOCX)

S2 Table Stratified analysis between (ΔPaO2/FiO2)/PEEP and in-hospital mortality. HR, hazard ratio; CI, confidence interval.
(DOCX)
