# Supplementary material for: Association between (ΔPaO2/FiO2)/PEEP and in-hospital mortality in patients with COVID-19 pneumonia: A secondary analysis
Source: PLoS One. 2024 May 31;19(5):e0304518. doi: 10.1371/journal.pone.0304518 (PMC11142544; doi:10.1371/journal.pone.0304518)
Supplement: S1 Table — IMV, invasive mechanical ventilation. These covariates produced over 10% change in the regression coefficient of (ΔPaO2/FiO2)/PEEP and were adjusted in multivariate analysis when added to the basic model or removed from the full model. (DOCX) [file pone.0304518.s001.docx]

**S1 Table The regression coefficient in the basic model and full model**

| covariates | β value in basic model | β value in full model | Choose? |
| --- | --- | --- | --- |
| Unadjusted β | -1.3713 | -4.2895 |  |
| Sex | -1.3692 | -3.9435 |  |
| Age | -1.3614 | -2.8370* | Yes |
| Obesity | -1.3849 | -3.4918* | Yes |
| Hypertension | -1.3652 | -4.2373 |  |
| Diabetes | -1.4521 | -3.7065* | Yes |
| Chronic renal insufficiency | -1.2333* | -4.3026 | Yes |
| Heart failure | -1.3376 | -4.2654 |  |
| Asthma | -1.3965 | -3.5852* | Yes |
| Immunosuppression | -1.4940 | -2.6518* | Yes |
| White blood cells (× 10^9^/L) | -1.3701 | -4.2056 |  |
| Lymphocytes (× 10^9^/L) | -1.3463 | -3.8647 |  |
| Platelets (×10^9^/L) | -1.3834 | -3.8934 |  |
| C-reactive protein (mg/L) | -1.1815* | -3.5506* | Yes |
| Procalcitonin (ng/mL) | -1.3654 | -2.9918* | Yes |
| Alanine aminotransferase (U/L) | -1.4109 | -1.9349* | Yes |
| Aspartate aminotransferase (U/L) | -1.3710 | -2.0072* | Yes |
| Creatinine phosphokinase-Total (U/L) | -1.2862 | -4.1057 |  |
| Creatinine phosphokinase-MB (U/L) | -1.3904 | -4.4556 |  |
| Lung damage on computed tomography | -1.2499 | -4.2136 |  |
| Sequential organ failure assessment | -1.5297* | -3.2801* | Yes |
| Corticosteroids | -1.3718 | -4.1436 |  |
| Colchicine | -1.3333 | -4.0483 |  |
| Tocilizumab | -1.3027 | -3.9730 |  |
| Renal replacement therapy | -1.4120 | -4.0714 |  |
| Sepsis | -1.2739 | -3.3976* | Yes |
| Septic shock | -1.2397 | -4.1605 |  |
| Acute kidney failure | -1.1748* | -4.1574 | Yes |
| Arrhythmia | -1.4221 | -3.9963 |  |
| Pneumonia associated with IMV | -1.5836* | -2.5121* | Yes |
| Catheter-associated bacteremia | -1.3437 | -4.2395 |  |
| Plateau pressure 24 h after IMV (cmH_2_O) | -1.3256 | -3.8922 |  |
| Driving pressure 24 h after IMV (cmH_2_O) | -1.3733 | -3.1711* | Yes |

IMV, invasive mechanical ventilation

These covariates produced over 10% change in the regression coefficient of (ΔPaO_2_/FiO_2_)/PEEP and were adjusted in multivariate analysis when added to the basic model or removed from the full model
