# Supplementary material for: Association between (ΔPaO2/FiO2)/PEEP and in-hospital mortality in patients with COVID-19 pneumonia: A secondary analysis
Source: PLoS One. 2024 May 31;19(5):e0304518. doi: 10.1371/journal.pone.0304518 (PMC11142544; doi:10.1371/journal.pone.0304518)
Supplement: S2 Table — HR, hazard ratio; CI, confidence interval. (DOCX) [file pone.0304518.s002.docx]

**S2 Table Stratified analysis between (ΔPaO_2_/FiO_2_)/PEEP and in-hospital mortality**

| **Exposure** | **Variable** | **HR(95% CI)** |
| --- | --- | --- |
|  | Age (years) |  |
| N |  | 200 |
| Low (< 11.80 ) | < 65 | 1.0 |
| High (≥ 11.80) | < 65 | 0.18 (0.08, 0.45) |
| Low (< 11.80 ) | ≥ 65 | 0.79 (0.38, 1.65) |
| High (≥ 11.80) | ≥ 65 | 0.66 (0.28, 1.51) |
| P for interaction |  | 0.026 |
|  | C-reactive protein (mg/L) |  |
| N |  | 190 |
| Low (< 11.80 ) | ≤ 100 | 1.0 |
| High (≥ 11.80) | ≤ 100 | 1.29 (0.37, 4.42) |
| Low (< 11.80 ) | > 100 | 3.63 (1.28, 10.31) |
| High (≥ 11.80) | > 100 | 0.61 (0.16, 2.26) |
| P for interaction |  | 0.007 |

HR, hazard ratio; CI, confidence interval
