# Supplementary material for: Association between (ΔPaO2/FiO2)/PEEP and in-hospital mortality in patients with COVID-19 pneumonia: A secondary analysis
Source: PLoS One. 2024 May 31;19(5):e0304518. doi: 10.1371/journal.pone.0304518 (PMC11142544; doi:10.1371/journal.pone.0304518)
Supplement: Attachment — Submitted filename: Response to Reviewers.docx [file pone.0304518.s003.docx]

Journal requirements:

When submitting your revision, we need you to address these additional requirements.

1. Please ensure that your manuscript meets PLOS ONE's style requirements, including those for file naming. The PLOS ONE style templates can be found at

https://journals.plos.org/plosone/s/file?id=wjVg/PLOSOne_formatting_sample_main_body.pdf and https://journals.plos.org/plosone/s/file?id=ba62/PLOSOne_formatting_sample_title_authors_affiliations.pdf

Reply: Thank you very much for your advice. We have accessed both of these URLs and the article formatting has been changed according to the style requirements.

2. Please amend either the title on the online submission form (via Edit Submission) or the title in the manuscript so that they are identical.

Reply: Thank you very much for your advice. We changed the titles in the online submission form and the manuscript and they are the same.

3. Your ethics statement should only appear in the Methods section of your manuscript. If your ethics statement is written in any section besides the Methods, please move it to the Methods section and delete it from any other section. Please ensure that your ethics statement is included in your manuscript, as the ethics statement entered into the online submission form will not be published alongside your manuscript.

Reply: Thank you very much for your advice. We have removed the code of ethics from all sections except the "Methods" section.

Additional Editor Comments:

Dear Authors:

Thank you for your recent submission.

Your research paper has now been evaluated by two experts, and we have discovered significant information that can contribute to the scientific community. However, there are some issues that need to be corrected so that we can reconsider your work further.

Please take the time to go over the reviewers' opinions as well as their recommendations. Please respond to the points that have been brought up and handle the paper in the manner that has been advised.

Also, please take care that .

The aforementioned statistical points must be rectified and corrected.

Given the limitations mentioned, the conclusion should be rewritten in both the abstract and at the end of the paper. It should also mention that further randomized, well-designed trials should be conducted to investigate the findings in order to make generalized recommendations.

Reply: Thank you very much for your advice. Your advice is very good. Our conclusion that “The (ΔPaO_2_/FiO_2_)/PEEP ratio was linearly associated with in-hospital mortality in patients with COVID-19 pneumonia. The (ΔPaO_2_/FiO_2_)/PEEP ratio can be used as a simple tool to evaluate patient prognosis and guide clinical management” was not be appropriate. We rewrote the conclusion as “The (ΔPaO_2_/FiO_2_)/PEEP ratio was associated with in-hospital mortality in patients with COVID-19 pneumonia” and deleted the sentence “The (ΔPaO_2_/FiO_2_)/PEEP ratio can be used as a simple tool to evaluate patient prognosis and guide clinical management”. Thank you again for your suggestion!

[Note: HTML markup is below. Please do not edit.]

Reviewers' comments:

Reviewer's Responses to Questions

Comments to the Author

1. Is the manuscript technically sound, and do the data support the conclusions?

The manuscript must describe a technically sound piece of scientific research with data that supports the conclusions. Experiments must have been conducted rigorously, with appropriate controls, replication, and sample sizes. The conclusions must be drawn appropriately based on the data presented.

Reviewer #1: Yes

Reviewer #2: Yes

2. Has the statistical analysis been performed appropriately and rigorously?

Reviewer #1: Yes

Reviewer #2: Yes

3. Have the authors made all data underlying the findings in their manuscript fully available?

The PLOS Data policy requires authors to make all data underlying the findings described in their manuscript fully available without restriction, with rare exception (please refer to the Data Availability Statement in the manuscript PDF file). The data should be provided as part of the manuscript or its supporting information, or deposited to a public repository. For example, in addition to summary statistics, the data points behind means, medians and variance measures should be available. If there are restrictions on publicly sharing data—e.g. participant privacy or use of data from a third party—those must be specified.

Reviewer #1: Yes

Reviewer #2: Yes

4. Is the manuscript presented in an intelligible fashion and written in standard English?

PLOS ONE does not copyedit accepted manuscripts, so the language in submitted articles must be clear, correct, and unambiguous. Any typographical or grammatical errors should be corrected at revision, so please note any specific errors here.

Reviewer #1: Yes

Reviewer #2: Yes

5. Review Comments to the Author

Please use the space provided to explain your answers to the questions above. You may also include additional comments for the author, including concerns about dual publication, research ethics, or publication ethics. (Please upload your review as an attachment if it exceeds 20,000 characters)

Reviewer #1: (ΔPaO2/FiO2)/PEEP seems to be a good predictor of outcome in patients with respiratory failure secondary to Covid-19. However, it is likely be a marker of disease severity rather than a modifiable risk factor. It is not clear from the manuscript what suggestions the authors have for clinicinas at bedside for modifying this risk factor. Was there any evidence in the data to suggest that the PEEP or FiO2 was not titrated appropriately?

Reply: Thank you very much for your advice. Your advice is very good. Our conclusion that “The (ΔPaO_2_/FiO_2_)/PEEP ratio can be used as a simple tool to evaluate patient prognosis and guide clinical management” was not be appropriate. We have deleted the sentence “The (ΔPaO_2_/FiO_2_)/PEEP ratio can be used as a simple tool to evaluate patient prognosis and guide clinical management”.

We also deleted the sentence “Therefore, (ΔPaO_2_/FiO_2_)/PEEP may help to better assess the risk of death.”

Following gramattical/typographical errors for the consideration of the authors

Line 167, Most commom complications should probably read as most common comorbidities

Reply: Thank you very much for your advice. Your advice is very good. We have modified our text as advised. “Hypertension and obesity were the most common comorbidities in patients with COVID-19 pneumonia”.

Line 169 - 172, could be reformated to draw the attention of the reader to the limitations of PaO2/FiO2 ratio

Reply: Thank you very much for your advice. Your advice is very good. We have added the sentence “The PaO_2_/FiO_2_ ratio had some limitations”.

Line 193 insufficiency are not known could read as insufficiency was not available in the primary dataset

Reply: Thank you very much for your advice. Your advice is very good. We have modified our text as advised. Due to the limitations of the secondary study, data on cardiac insufficiency was not available in the primary dataset.

Reviewer #2: I have gone through the study and have found some merit. But there are some suggestions and points which must be accommodated before the final acceptance of study.

1. The authors have presented Hazard Ratio in the results section of abstract with their confidence intervals but its missing p-value. p-value increases the worth of results. For each HR the associated p-value must be mentioned.

Reply: Thank you very much for your advice. Your advice is very good. We have modified our text as advised. In-hospital mortality was lower in the high (ΔPaO_2_/FiO_2_)/PEEP group than in the low (ΔPaO_2_/FiO_2_)/PEEP group [18 (13%) vs. 38 (38%)]; hazard ratio (HR), 0.33 [95% confidence intervals (CI), 0.17-0.61, P < 0.001], adjusted HR, 0.32 (95% CI, 0.11-0.94, P = 0.038). After adjusting for confounding variables, we found that each unit increase in (ΔPaO_2_/FiO_2_)/PEEP was associated with a 12% reduction in the risk of in-hospital mortality [HR, 0.88, 95%CI, 0.80-0.97, P = 0.013)].

2.The conclusion section of abstract is unclear. What is meant by linearly associated? On what grounds the authors declared it linearly associated. It will be better if conclusion is general not specific about relationship.

Reply: Thank you very much for your advice. Your advice is very good. We have deleted the word "linearly". The (ΔPaO_2_/FiO_2_)/PEEP was associated with in-hospital mortality in patients with COVID-19 pneumonia.

3.In line 117,"Their mean age was 54.29 years, and 42 patients (21.0%) were female". This line should be separated into two lines explaining about mean age with SD. The numbers and associated percentages should be mentioned for both male and female.

Reply: Thank you very much for your advice. Your advice is very good. We have modified our text as advised. The mean age of patients was 54.29 ± 12.20 years. Among 200 patients, 42 (21%) were female and 158 (79%) were male.

4. From line 117-125, the authors discussed the significant difference but did not mention the p-values or any result with the statement supporting their outcomes. It will better if significant or non-significant results be mentioned with each statement

Reply: Thank you very much for your advice. Your advice is very good. We have modified our text as advised. “There was a statistical difference in comorbidities between the two groups, with chronic kidney disease (CKI) and heart failure being more common comorbidities in the low (ΔPaO_2_/FiO_2_)/PEEP group (chronic kidney disease, 8% vs. 0, P = 0.007; heart failure, 10% vs. 0, P = 0.001). Lung damage on computed tomography and platform pressure levels were higher in the low (ΔPaO_2_/FiO_2_)/PEEP group (lung damage on computed tomography > 50%, 73% vs. 52%, P = 0.002; platform pressure levels > 30 cmH2O, 33% vs. 20%, P = 0.037). Sepsis, septic shock, acute kidney failure, and renal replacement therapy were also more common in the low (ΔPaO_2_/FiO_2_)/PEEP group (sepsis, 80% vs 63%, P = 0.008; septic shock, 37% vs. 21%, P = 0.013; acute kidney failure, 22% vs. 7%, P = 0.003, renal replacement therapy, 12% vs. 3%, P = 0.016). We found that PaO_2_/FiO_2_ after 24 h of IMV were lower and PEEP was higher in the low (ΔPaO_2_/FiO_2_)/PEEP group compared with the high (ΔPaO_2_/FiO_2_)/PEEP group (197.85 ± 55 vs. 301.49 ± 71.75, P < 0.001). There was no difference in PaO_2_/FiO_2_ before entering IMV between the two groups (100.65 ± 43.62 vs. 99.81 ± 34.18, P = 0.880).”

5.In whole study, authors some time used past tense and sometimes present tense. It should be adjusted for the complete study and should have same pattern for complete study. In discussion, authors used present tense while after few lines past tense were used. It should be checked properly.

Reply: Thank you very much for your advice. Your advice is very good. We have modified our text as advised.

1) Studies ~~have~~ reported that a proportion of COVID-19 patients (23.6% to 33.1%) required IMV.

2) The arterial pressure of oxygen (PaO_2_)/inspiratory fraction of oxygen (FiO_2_) ~~is~~ was both an important indicator of tracheal intubation and a correlate of mortality.

3) Previous studies ~~have~~ focused on the relationship between PaO_2_/FiO_2_ and prognosis at the time of admission to the hospital or intensive care unit (ICU).

4) Their studies ~~have shown~~ showed that lower PaO_2_/FiO_2_ ~~is~~ was associated with increased in-hospital mortality in COVID-19 patients.

5) Therefore, PaO_2_/FiO_2_ ~~may~~ might not be a good assessment of prognosis in COVID-19 patients requiring IMV.

6) However, the value of PaO_2_ ~~can~~ could be influenced by end-expiratory pressure (PEEP), and patients with higher PEEP ~~may~~ might have more severe lung injury than those with lower PEEP for the same ΔPaO_2_/FiO_2_.

7) This ~~is~~ was a secondary analysis study.

8) Mechanical ventilation management strategies and prone position ventilation management strategies for this cohort ~~have been~~ were described in full previously.

9) Demographic and clinical characteristics of patients ~~are~~ were shown in Table 1 according to (ΔPaO_2_/FiO_2_)/PEEP.

10) The Kaplan-Meier curves for survival rate ~~are~~ were shown in Fig 1.

11) In this study, the median duration of mechanical ventilation was 10 days, the median PEEP was 12, and the mortality rate was 25%, which ~~is~~ was consistent with previous studies (14, 15). Hypertension and obesity were the most common comorbidities of COVID-19, and the majority of patients were male, which ~~is~~ was also consistent with previous studies (16).

12) In this study, the median duration of mechanical ventilation was 10 days, the median PEEP was 12, and the mortality rate was 25%, which ~~is~~ was consistent with previous studies (14, 15).

13) Hypertension and obesity were the most common comorbidities of COVID-19, and the majority of patients were male, which ~~is~~ was also consistent with previous studies (16).

14) The PaO_2_/FiO_2_ ratio had some limitations. In patients requiring IMV, PaO2/FiO2 values could be ~~are~~ overestimated on admission to the hospital or ICU (7).

15) On the other hand, PaO_2_/FiO_2_ values before IMV ~~may~~ might be underestimated in emergency situations such as sputum occlusion and respiratory arrest (17).

16) In addition, the mode of oxygen administration before tracheal intubation, such as nasal cannula oxygenation, mask oxygenation, high-flow oxygen therapy, and noninvasive ventilator-assisted ventilations, ~~may~~ might also affect the pre-mechanical ventilation PaO_2_/FiO_2_ values (18).

17) Patients with significant changes in the difference in PaO_2_/FiO_2_ before and after intubation (ΔPaO_2_/FiO_2_) ~~may~~ might not have diffuse alveolar damage (19).

18) Patients requiring higher PEEP to prevent recurrent alveolar collapse ~~may~~ might have combined more severe lung damage (20).

19) Previous studies showed ~~It has been reported~~ that a high percentage of COVID-19 patients admitted to ICU developed cardiac systolic and diastolic dysfunction (21-23).

20) Although higher PEEP values helped to improve oxygenation, high PEEP values could ~~can~~ also lead to impaired right ventricular and hemodynamic function (24).

21) Patients might ~~may~~ require additional fluid and vasopressors.

22) Since higher values of PEEP ~~have an impact on~~ could affect cardiac function (25), this might ~~may~~ partly explain the higher mortality in patients with low (ΔPaO_2_/FiO_2_)/PEEP group.

23) To achieve the prone ventilation goal, patients ~~may~~ might require high regimens of sedatives, analgesics, and neuromuscular blocking agents.

24) Deep sedation in COVID-19 patients ~~may~~ might be associated with several complications such as ventilation-associated pneumonia, prolonged MV duration, and ICU-acquired weakness (26).

25) These complications ~~may~~ might further lead to prolonged ICU and hospitalization and increased mortality (26, 27).

26) This study ~~has~~ had some limitations.

27) Thus, the findings ~~may~~ might not be generalizable to the general population.

28) Fourth, although we adjusted for possible confounding variables, other unmeasured variables ~~may~~ might affect the results (28).

6.The limitation of the study should be separated from the discussion section.

Reply: Thank you very much for your advice. Your advice is very good. We have modified our text as advised.

7. The conclusion section is short.It should be expanded and should discuss other aspects of study also like theoretical and practical implications.

Reply: Thank you very much for your advice. Your advice is very good. We have modified our text as advised.

The (ΔPaO_2_/FiO_2_)/PEEP ratio was associated with in-hospital mortality in patients with COVID-19 pneumonia. (ΔPaO_2_/FiO_2_)/PEEP might be a marker of disease severity in COVID-19 patients.

8. In table 1, p-value should accompany the test-statistic value also.

Reply: Thank you very much for your advice. Your advice is very good. We have modified our text as advised.

| **Variable** | **Low (< 11.80)** | **High (≥ 11.80)** | **χ^2^** | **p*-*Value** |
| --- | --- | --- | --- | --- |
| Number | 100 | 100 |  |  |
| Female | 20 (20%) | 22 (22%) | 0.121 | 0.728 |
| Age (year) |  |  | 0.030 | 0.861 |
| < 65 | 79 (79%) | 80 (80%) |  |  |
| ≥ 65 | 21 (21%) | 20 (20%) |  |  |
| Obesity | 61 (61%) | 57 (57%) | 0.331 | 0.565 |
| Hypertension | 31 (31%) | 22 (22%) | 2.079 | 0.149 |
| Diabetes | 22 (22%) | 21 (21%) | 0.030 | 0.863 |
| Chronic renal insufficiency | 8 (8%) | 0 (0) | - | 0.007 |
| Heart failure | 10 (10%) | 0 (0) | 10.526 | 0.001 |
| Asthma | 12 (12%) | 18 (18%) | 1.142 | 0.235 |
| Immunosuppression | 11 (11%) | 7 (7.07%) | 0.934 | 0.334 |
| White blood cells (×10^9^/L) |  |  | 0.731 | 0.393 |
| ≤ 10 | 53 (53%) | 59 (59%) |  |  |
| > 10 | 47 (47%) | 41 (41%) |  |  |
| Lymphocytes (×10^9^/L) |  |  | 0.026 | 0.873 |
| ≤ 1 | 73 (73%) | 74 (74%) |  |  |
| > 1 | 27 (27%) | 26 (26%) |  |  |
| Platelets (×10^9^/L) |  |  | 2.922 | 0.087 |
| ≤ 300 | 62 (62%) | 50 (50%) |  |  |
| > 300 | 38 (38%) | 50 (50%) |  |  |
| C-reactive protein (mg/L) |  |  | 1.027 | 0.311 |
| ≤ 100 | 32 (33.33%) | 38 (40.43%) |  |  |
| > 100 | 64 (66.67%) | 56 (59.57%) |  |  |
| Procalcitonin (ng/mL) |  |  | 3.128 | 0.077 |
| ≤ 0.5 | 55 (75.34%) | 55 (87.30%) |  |  |
| > 0.5 | 18 (24.66%) | 8 (12.70%) |  |  |
| Alanine aminotransferase (U/L) |  |  | 0.226 | 0.635 |
| ≤ 40 | 26 (26%) | 29 (29%) |  |  |
| > 40 | 74 (74%) | 71 (71%) |  |  |
| Aspartate aminotransferase (U/L) |  |  | 0.546 | 0.460 |
| ≤ 40 | 33 (33%) | 38 (38%) |  |  |
| > 40 | 67 (67%) | 62 (62%) |  |  |
| Creatinine phosphokinase-Total (U/L) |  |  | 1.602 | 0.206 |
| ≤ 200 | 59 (62.77%) | 63 (71.59%) |  |  |
| > 200 | 35 (37.23%) | 25 (28.41%) |  |  |
| Creatinine phosphokinase-MB (U/L) |  |  | 0.078 | 0.780 |
| ≤ 25 | 45 (46.88%) | 45 (48.91%) |  |  |
| > 25 | 51 (53.12%) | 47 (51.09%) |  |  |
| Lung damage on computed tomography |  |  | 9.408 | 0.002 |
| ≤ 50% | 27 (27%) | 48 (48%) |  |  |
| > 50% | 73 (73%) | 52 (52%) |  |  |
| Sequential organ failure assessment |  |  | 1.962 | 0.161 |
| ≤ 4 | 61 (61.62%) | 71 (71%) |  |  |
| > 4 | 38 (38.38%) | 29 (29%) |  |  |
| Antibiotics | 99 (99%) | 98 (98%) | - | 1 |
| Corticosteroids | 98 (98%) | 96 (96.97%) | - | 0.683 |
| Colchicine | 32 (32%) | 20 (20%) | 3.742 | 0.053 |
| Tocilizumab | 14 (14%) | 12 (12%) | 0.177 | 0.674 |
| Renal replacement therapy | 12 (12%) | 3 (3%) | 5.834 | 0.016 |
| Sepsis | 80 (80%) | 63 (63%) | 7.091 | 0.008 |
| Septic shock | 37 (37%) | 21 (21%) | 6.217 | 0.013 |
| Acute kidney failure | 22 (22%) | 7 (7%) | 9.074 | 0.003 |
| Arrhythmia | 9 (9.09%) | 4 (4%) | 2.112 | 0.146 |
| Pneumonia associated with IMV | 26 (26%) | 18 (18%) | 1.865 | 0.172 |
| Catheter-associated bacteremia | 6 (6%) | 5 (5%) | 0.096 | 0.756 |
| Plateau pressure 24 h after IMV (cmH_2_O) |  |  | 4.338 | 0.037 |
| ≤ 30 | 67 (67%) | 80 (80%) |  |  |
| > 30 | 33 (33%) | 20 (20%) |  |  |
| Driving pressure 24 h after IMV (cmH_2_O) |  |  | 1.025 | 0.311 |
| ≤ 15 | 36 (36%) | 43 (43%) |  |  |
| > 15 | 64 (64%) | 57 (57%) |  |  |
| Tidal volume 24 h after IMV (ml) | 459.47 ± 82.28 | 474.27 ± 72.53 | t = -1.349 | 0.179 |
| PaO_2_/FiO_2_ before IMV (mmHg) | 100.65 ± 43.62 | 99.81 ± 34.18 | t = 0.151 | 0.880 |
| PaO_2_/FiO_2_ 24 h after IMV (mmHg) | 197.85 ± 55 | 301.49 ± 71.75 | t = -11.464 | <0.001 |
| PEEP 24 h after IMV (cmH_2_O) | 12.70 ± 2 | 11.69 ± 1.65 | t = 3.893 | <0.001 |
| IMV LOS (days) | 11 (7-19) | 8.50 (5-13.25) | z = -2.297 | 0.023 |
| ICU LOS (days) | 11 (7-17.25) | 9 (5 - 14) | z = -1.886 | 0.061 |
| Hospital LOS (days) | 20 (15-28) | 20 (13-29) | z = -0.223 | 0.822 |
| In-hospital mortality | 38 (38%) | 13 (13%) | 16.450 | <0.001 |

9.The authors mentioned in their study that Continuous data were presented as mean ± standard deviation (SD) if normally distributed. Which variables were tested normal and which were non-normal. If all the variables were non-normal then this statement should be excluded. it would create confusion between normality and non-normality issue. the statement can be "Continuous data were presented as mean ± standard deviation (SD)".

Reply: Thank you very much for your advice. Your advice is very good. We have modified our text.

Tidal volume 24 h after IMV, PaO_2_/FiO_2_ before IMV, PaO_2_/FiO_2_ 24 h after IMV, and PEEP 24 h after IMV (cmH_2_O) were normally distributed.

IMV LOS, ICU LOS, and Hospital LOS were non-normally distributed.

6. PLOS authors have the option to publish the peer review history of their article (what does this mean?). If published, this will include your full peer review and any attached files.

Reply：Yes.

If you choose “no”, your identity will remain anonymous but your review may still be made public.

Do you want your identity to be public for this peer review? For information about this choice, including consent withdrawal, please see our Privacy Policy.

Reviewer #1: Yes: Kesava Ananth Ramakrishnan

Reviewer #2: No

[NOTE: If reviewer comments were submitted as an attachment file, they will be attached to this email and accessible via the submission site. Please log into your account, locate the manuscript record, and check for the action link "View Attachments". If this link does not appear, there are no attachment files.]

While revising your submission, please upload your figure files to the Preflight Analysis and Conversion Engine (PACE) digital diagnostic tool, https://pacev2.apexcovantage.com/. PACE helps ensure that figures meet PLOS requirements. To use PACE, you must first register as a user. Registration is free. Then, login and navigate to the UPLOAD tab, where you will find detailed instructions on how to use the tool. If you encounter any issues or have any questions when using PACE, please email PLOS at figures@plos.org. Please note that Supporting Information files do not need this step.

Reply: Dear Editor, Thank you very much for your suggestion. We have registered for PACE and put the images on the PACE software for editing.
